# Peroxisome Proliferator-Activated Receptors in Female Reproduction and Fertility

**DOI:** 10.1155/2016/4612306

**Published:** 2016-07-31

**Authors:** Maurizio Vitti, Giovanna Di Emidio, Michela Di Carlo, Gaspare Carta, Andrea Antonosante, Paolo Giovanni Artini, Annamaria Cimini, Carla Tatone, Elisabetta Benedetti

**Affiliations:** ^1^Department of Life, Health and Environmental Sciences, University of L'Aquila, 67100 L'Aquila, Italy; ^2^Gynecology and Fertility Unit, San Salvatore Hospital, 67100 L'Aquila, Italy; ^3^Department of Experimental and Clinical Medicine, Division of Gynecology and Obstetrics, University of Pisa, Pisa, Italy; ^4^Sbarro Institute for Cancer Research and Molecular Medicine and Center for Biotechnology, Temple University, Philadelphia, PA, USA; ^5^National Institute for Nuclear Physics (INFN), Gran Sasso National Laboratory (LNGS), Assergi, Italy

## Abstract

Reproductive functions may be altered by the exposure to a multitude of endogenous and exogenous agents, drug or environmental pollutants, which are known to affect gene transcription through the peroxisome proliferator-activated receptors (PPARs) activation. PPARs act as ligand activated transcription factors and regulate metabolic processes such as lipid and glucose metabolism, energy homeostasis, inflammation, and cell proliferation and differentiation. All PPARs isotypes are expressed along the hypothalamic-pituitary-gonadal axis and are strictly involved in reproductive functions. Since female fertility and energy metabolism are tightly interconnected, the research on female infertility points towards the exploration of potential PPARs activating/antagonizing compounds, mainly belonging to the class of thiazolidinediones (TZDs) and fibrates, as useful agents for the maintenance of metabolic homeostasis in women with ovarian dysfunctions. In the present review, we discuss the recent evidence about PPARs expression in the hypothalamic-pituitary-gonadal axis and their involvement in female reproduction. Finally, the therapeutic potential of their manipulation through several drugs is also discussed.

## 1. PPARs

Peroxisome proliferator-activated receptors (PPARs) are ligand activated transcription factors belonging to the nuclear receptor family; three isotypes have been identified so far: PPAR*α*, PPAR*β*/*δ*, and PPAR*γ*, from frogs to humans [1]. A multitude of agents can bind the PPARs, influencing their activity. Endogenous compounds include arachidonic acid and its eicosanoid metabolites [2–4], while synthetic ligands are principally represented by fibrates (hypolipidemic drugs) [5], thiazolidinediones (TZDs) (antidiabetic drugs) [6], and nonsteroidal anti-inflammatory drugs [7]. PPARs can be also activated by environmental pollutants such as certain phthalate esters (plasticizers), herbicides, and organic solvents [8]. The binding specificity of these compounds towards PPAR subtypes is not strict; that is, several prostaglandins activate all three PPAR subtypes, while most fibrates and TZDs selectively bind to PPAR*α* and PPAR*γ*, respectively [9]. After ligand binding, PPARs undergo a conformational change resulting in the dissociation of corepressors [10, 11] and recruitment of coactivators [11]. PPARs heterodimerize with the 9-cis-retinoic acid X receptor (RXR, NR2B) to form PPAR/RXR complex that binds to a specific DNA sequence called PPAR-responsive element (PPRE), usually 5′-AACT AGGNCA A AGGTCA-3′ [10]. The PPRE is usually located in the promoter region of key target genes mainly involved in lipid and cholesterol metabolism [10, 12, 13], cell differentiation [14, 15], organogenesis [16], antiangiogenic activity [17], and insulin sensitization [18]. The PPAR family is known to adapt cell metabolism to lipid/glucose availability by controlling energy homeostasis genes. All three PPAR isotypes regulate endocrine pathways.

PPARs are expressed in the female hypothalamic-pituitary-gonadal axis and they act on critical processes for ovarian function. For instance, PPARs/RXRs may inhibit transactivation of the estrogen receptor through competition for ERE binding [19]. It is known in porcine ovarian follicles that PPAR*γ* can downregulate aromatase expression through the suppression of NF-*κ*B pathway [20] and that its synthetic activator rosiglitazone is able to stimulate PPAR*γ* expression affecting the expression of important enzymes involved in steroidogenesis [21]. Regarding PPAR*α*,* in vivo* experiments in mouse ovaries showed that fenofibrate inhibits gene expression of enzymes involved in ovarian estrogen synthesis and that a functional PPAR*α* is indispensable for this inhibitory action [22].

All the three PPAR isotypes regulate gametogenesis, ovulation, corpus luteum regression, and the implantation process [1, 23]. Regarding male gametogenesis mRNAs encoding PPAR*α*, PPAR*β*/*δ*, and PPAR*γ* are developmentally expressed in both differentiating germ and Sertoli cells; particularly, PPAR*γ* regulates the pattern of expression of key lipid metabolic genes in Sertoli cells [24].

*In situ* hybridization studies on rat ovary collected during follicular development and periovulatory period, by Komar and colleagues [25], demonstrated that PPAR*γ* mRNA is localized primarily into granulosa cells and that its expression does not change during follicular development; in contrast the treatment of animals with human CG (hCG) leads after 24 h to a not uniform decrease of PPAR*γ*. Particularly, at 24 h after hCG the levels of PPAR*γ* mRNA appeared reduced of 64%, but some follicles maintained high expression. PPAR*α* and PPAR*β*/*δ* mRNA levels, mainly present in theca and stroma cells, do not change even upon treatment.* In vitro* study confirmed that PPAR*γ* is involved in follicular development, since, in granulosa cells from PMSG-primed rats cultured for 48 h with PPAR*γ* ligands, an increase of progesterone and E2 secretion is observed [25].

The effect of PPAR*γ* on progesterone synthesis depends on cell type, stage of cell differentiation, stage of the ovarian cycle, and/or animal species [26].

For instance, the role of PPAR*γ* was investigated in* corpora lutea* of pseudopregnant rabbits at early, middle, and late stage. Both mRNA and protein levels of PPAR*γ* decreased from the early to the late stage.* In vitro* studies on* corpora lutea* treated with PPAR*γ* agonist show that the agonist is able to increase progesterone secretion and 3-beta-hydroxysteroid dehydrogenase (3-beta-HSD) activity at early and mid luteal stages, while decreasing at the same stages prostaglandin-endoperoxide synthase 2 (PTGS2) activity and prostaglandin F2a. Treatments with a specific antagonist of PPAR*γ* have opposite effects [27]. Quantitative analysis of PPARs mRNA in porcine endometrium through the estrous cycle and early pregnancy showed the presence of all three PPARs in this tissue. Particularly, this analysis showed a marked increase of PPAR*γ* mRNA level on days 13–15 of the estrous cycle and the decrease of PPAR*β*/*δ* on days 11-12 of pregnancy suggesting that PPARs are engaged, respectively, in luteolysis (*corpus luteum* regression) and maternal recognition of pregnancy in the pig [28]. In addition, PPAR ligands affect progesterone (P4) and 17b-estradiol (E2) secretion by porcine* corpus luteum* during pregnancy [29].

PPAR*β*/*δ* seems to play an important role in embryo implantation; in fact, several lines of evidence suggest that the effects of PGI2, the primary PG essential for implantation and decidualization, are mediated by PPAR*β*/*δ* [30]. Moreover, using molecular, pharmacologic, and genetic approaches, Kang and colleagues, 2001, showed that PGI2-induced PPAR*β*/*δ* activation accelerates blastocyst hatching in mice [31].

Furthermore, PPARs expression can be modulated by gonadotropin activity; that is, PPAR*γ* mRNA and protein levels are tightly regulated in the ovary by luteinizing hormone (LH) in rat [32] and in rhesus monkey granulosa cells [33]. Particularly, the data obtained in primates showed that one of the initial actions of LH/CG on preovulatory follicle is to rapidly reduce PPAR*γ* expression and its target gene NR1H3, enzyme that promotes the expression of periovulatory genes, such as* SCARB1* and* STAR* [33].

Moreover, gene array study conducted on human cumulus cells has revealed that PPAR*α* is among the genes differentially expressed when LH is supplemented to FSH during* in vitro* fertilization [34].

Recently we found differential effects of controlled ovarian stimulation COS protocols on PPARs and their steroidogenic targets in relation to LH and gonadotropin source. Particularly, the analyses of gene and protein expression of PPAR*α*, PPAR*β*/*δ*, and PPAR*γ* have revealed that r-hLH associated with r-hFSH exposure influence all the three PPARs by modulating the relative abundance of the different isotypes. In particular, a significant reduction of PPAR*α* protein was observed, although mRNA levels did not significantly change. PPAR*β*/*δ* and PPAR*γ* were significantly upregulated upon r-hFSH/r-hLH treatment [35].

Finally, PPARs/RXRs, as exposed above, may inhibit transactivation of the estrogen receptor through competition for ERE binding mediating also the effects of the endocrine disruptors, many of which are PPAR ligands. These events may lead to infertility as demonstrated in many aquatic species and also in rodents. In fact, in several species, PPARs are expressed in somatic cells and germ cells of the ovary as well as the testis. Invalidation of these receptors in mice or stimulation of these receptors* in vivo* or* in vitro* showed that each receptor has physiological roles in the gamete maturation or the embryo development. In addition, synthetic PPAR*γ* ligands are recently used to induce ovulation in women with polycystic ovary disease. These results reveal the positive actions of PPAR in reproduction. On the other hand, xenobiotics molecules (in herbicides, plasticizers, or components of personal care products), capable of activating PPAR, may disrupt normal PPAR functions in the ovary or the testis and have consequences on the quality of the gametes and the embryos [1].

## 2. Female Reproductive System

The female reproductive system requires precise coordination among the hypothalamic gonadotropin-releasing hormone (GnRH), the pituitary gonadotropins, the ovaries, and the uterus. The pituitary gland responds to GnRH pulses by releasing two gonadotropins: follicle stimulating hormone (FSH) and luteinizing hormone (LH). The FSH activity during the early-midfollicular phase is critical for recruitment and maturation of ovarian follicles, expression of LH receptors, and differentiation of an endocrinologically active theca layer capable of synthesizing androgens in response to LH [36]. Androgens are then aromatized to estradiol by the granulosa cells. The increase of estradiol and ovarian production of the inhibins, peptides that inhibit pituitary gland synthesis and secretion of FSH, reduces FSH levels, enhances LH responsiveness, and induces the LH surge [37]. LH levels remain increased for 36–48 hours and ovulation occurs 16 to 20 hours after the peak of LH surge. Resumption of meiosis and progression to the second meiotic metaphase and polar body extrusion take place just prior to ovulation. After the release of the oocyte-cumulus complex, estradiol levels decline and luteinization of the follicle results in increased production of progesterone [38]. The rise in progesterone levels induces a significant decrease in GnRH/LH pulse frequency and prepares the genital tract for a possible embryo implantation by increasing secretory activity of the endometrial glands and by changing composition of the cervical mucus that becomes thick and viscous [39]. Therefore, the ovarian cycle requires a fine coordination of the hypothalamic-pituitary-gonadal axis and follicular growth to drive steroidogenesis that culminates in the ovulation of a fully competent oocyte. Altered secretion of gonadotropins and poor ovarian steroidal response are associated with reproductive dysfunctions [40, 41] characterizing a large percentage of women who undergo* in vitro* fertilization (IVF) treatments. In IVF, the gonadotropin therapy plays a pivotal role in ovarian stimulation and influences egg retrieval, fertilization, embryo culture, and intrauterine transfer [42].

## 3. The Role of PPARs in Female Reproductive Function

**Figure 1:**
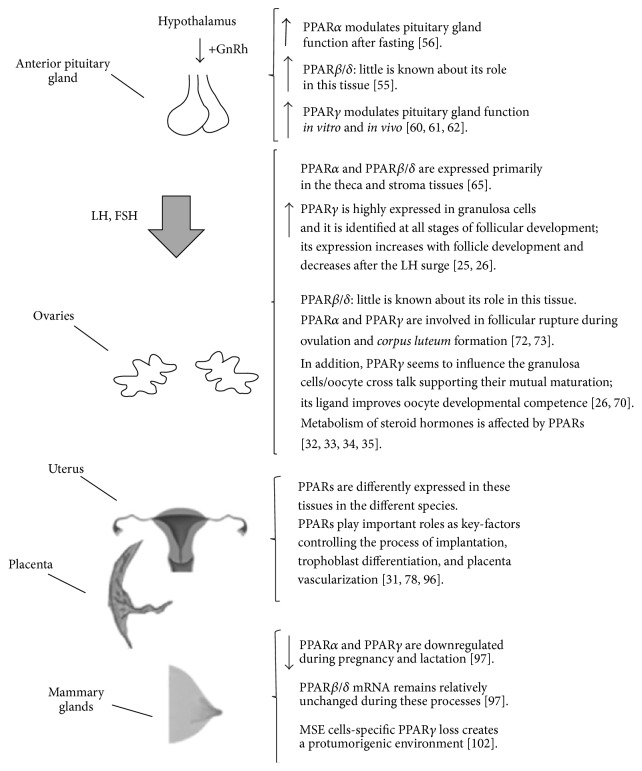
PPARs expression and role in the whole female reproductive system.

A multitude of studies have revealed that PPARs are functionally expressed in the whole female reproductive system indicating that PPARs may play an important role in reproduction mostly due to their implication in energy homeostasis (Figure 1). Moreover, a role for PPARs has been evoked in ovarian dysfunctions related to obesity [43, 44], dyslipidemia [45, 46], hyperandrogenemia [47–50], and insulin resistance [51–54].

### 3.1. Hypothalamic-Pituitary Axis

All PPAR isotypes are expressed in the mouse pituitary gland [55]; recent evidence in rats has demonstrated that fasting increases mRNA levels of PPAR*α* targets implicated in *β*-oxidation of fatty acids, such as acyl-CoA oxidase, carnitine palmitoyltransferase-1, medium chain acyl-CoA dehydrogenase, and ketogenesis, that is, mitochondrial 3-hydroxy-3-methylglutaryl-CoA synthase in the pituitary gland. Furthermore, studies on PPARa* null* mice have revealed that PPAR*α* increases pituitary production of prolactin and LH-beta genes upon fasting [56].

PPAR*γ* is known to be highly expressed in normal human pituitary gland [57] and in the rat hypothalamus [58, 59]. It was reported that PPAR*γ* activation by TZDs prevented the development of pituitary adenomas in mice and humans due to its antiproliferative effects, so TZDs are proposed as novel oral therapy for pituitary tumors [60]. PPAR*γ* expression and function in the hypothalamopituitary-ovary axis in the sheep were studied by Froment and colleagues, 2003. They found that PPAR*γ* is expressed in the pituitary gland but not in the hypothalamus and through transient transfection experiments on pituitary cells with a vector containing PPREs driving the firefly luciferase gene (PPRE-Luc); they found a small but significant effect of rosiglitazone on endogenous pituitary PPAR*γ*, indicating that it is functional and could play a physiological role in this tissue [61]. Since the precise role of PPAR*γ* in regulating the hypothalamic-pituitary-gonadal axis remains unclear, recently Sharma and colleagues, 2011, had tried to dissect the role of this transcriptional factor in the pituitary gland [62]. They showed that rosiglitazone downregulates GnRH-mediated phosphorylation of the JNKs and p38MAPKs and Lhb and Fshb gene expression in L*β*T2 cells. Thus, the PPAR*γ* activation could act by reducing the basal activity of the gonadotropin gene promoters. Since these kinases are activated by many cytokines and inflammatory signals, the authors hypothesize that the TZD effect is consistent with its known anti-inflammatory actions. The same authors moreover showed that pituitary gland cre* PPARg*-knockout (PKO) mice have elevated plasma LH and reduced litter size compared to the control cre-negative littermates. Female PKO mice present also fertility defects like the transgenic mice with 10- to 12-fold overexpression of a stabilized form of LH. These observations support a possible role for PPAR*γ* in modulating pituitary gland function* in vitro* and* in vivo*.

As regards PPAR*β*/*δ* very little is known about its role in this tissue.

### 3.2. Ovary

The basic unit of the ovary is the follicle, a functional syncytium formed by the oocyte and surrounding follicle cells. During reproductive life, a number of oocytes from the pool of quiescent follicles progress to mature and ovulate. Once recruited in the growing phase, primordial follicles develop into primary, secondary, and eventually antral follicle reaching the preovulatory stage under gonadotropin stimulation. The antral follicle is characterized by the presence of an antrum filled with follicular fluid bathing an oocyte surrounded by cumulus cells. The inner lining of the follicle is formed by granulosa cells, which are principally responsible for the androgens-estrogens conversion. The outer boundary is defined by theca cells, which form a vascularized cell layer that provides androgens to granulosa cells. Upon the LH surge, the dominant follicle ovulates the mature oocyte that is ready for fertilization, whereas the remaining theca and granulosa cells become the corpus luteum that contributes to the production of circulating progesterone [63, 64]. All PPAR isotypes have been observed in the ovarian tissue. PPAR*α* and PPAR*β*/*δ* are expressed primarily in the theca and stroma tissues [65]. PPAR*γ* expression has been reported in the ovaries of mouse, rat, pig, sheep, cow, and humans, with higher levels in granulosa cells and lower levels in theca cells and corpus luteum of rodents and ruminants [66]. In particular, PPAR*γ* has been identified in follicles at all stages of development and it has been shown that levels increase during follicle growth and decrease after the LH surge [25, 26]. In the rat, PPAR*γ* levels are lower in newly forming luteal tissue when compared with that in luteal tissue from previous ovulation [67]. PPAR*γ* has also been identified in oocytes from cattle [68] and zebrafish [69], suggesting an evolutionary conserved role for this isotype in female germ cells. Since PPAR*γ* expression is predominant in granulosa cells, it can be hypothesized that its activity influences the granulosa cell/oocyte cross talk, supporting their mutual maturation [26]. In this respect, some studies have investigated the possible effects of PPAR*γ* activation on oocyte maturation.* In vivo* administration of PPAR*γ* agonist rosiglitazone in diet-induced obese mice significantly improves oocyte competence supporting the conclusion that PPAR*γ* positively affects ovarian functions [70]. However direct exposure to rosiglitazone does not produce the same effects in cumulus-oocyte complexes from normal mice. In this experimental system, although oocyte mitochondrial activity increases, fatty acid metabolism decreases and subsequent impairment of embryo development was observed [71]. The discrepancy between these studies may be explained by considering that beneficial effects of PPAR*γ* activation in obese mice may result from systemic effects such as reduced circulating insulin and lipid. In addition it can be hypothesized that only oocytes with large lipid availability as those from obese mice may benefit from increased PPAR*γ* activity. These observations may be useful for investigating potential ability of PPAR*γ* agonists to improve oocyte competence following* in vitro* maturation in obese females. Finally, several proteases involved in follicular rupture during ovulation and corpus luteum formation, such as matrix metalloprotease-9, plasminogen activator, and plasminogen activator inhibitor, are regulated by PPAR*α* and PPAR*γ*  [72, 73]. In contrast, very little is known about the role of PPAR*β*/*δ* in this tissue.

Regarding ovarian function, it is well documented that the synthesis and metabolism of steroid hormones are affected by all PPARs. PPAR*α* positively regulates 17*β*-hydroxysteroid dehydrogenase IV (17 *β*-HSD IV), the enzyme that catalyzes the conversion of 17*β*-estradiol to estrone, its inactive form [74]. Although PPAR*α* expression is very low in granulosa cells [25, 67], it was demonstrated that its activation is responsible for decreasing the expression and activity of aromatase [22]. In addition, dietary supplementation of dehydroepiandrosterone (DHEA), a PPAR*α* ligand and precursor of androstenedione, has recently emerged as a beneficial intervention for increasing oocyte quality in poor responders and aged women [75, 76]. According to a model proposed by Ford [75], DHEA action may involve the activation of PPAR*α* target genes known to be critical for mitochondria functions. Thus, upregulation of PPAR*α* in the follicle microenvironment may support oxidative energy metabolism known to be jeopardized during physiological or pathological ovarian aging [77]. Moreover, the beneficial action by DHEA throughout PPAR*α* activation could be also ascribed to diminished levels of ceramide, a molecule involved in increased levels of apoptosis in aged follicles [75]. Furthermore, a gene array study conducted on human cumulus cells has revealed that PPAR*α* and RXR*β* were among the genes differentially expressed when LH is supplemented to FSH during IVF [34], thus highlighting their sensitivity to gonadotropins and suggesting their important role in follicle development as well as in steroidogenesis. In a recent study by our research group, we investigated the impact of different ovarian stimulation regimens based on r-hFSH alone, r-hFSH/r-hLH, or highly purified human menopausal gonadotropin (hMG-HP) on gene and protein expression of PPAR isotypes and their targets relevant to steroid metabolism, such as 3-hydroxy-3-methylglutaryl coenzyme A reductase (HMG-CoA red), cytochrome P-450 (CYP19A1), 17*β*-hydroxysteroid dehydrogenase type IV (17*β*HSD IV), and 3*β*-hydroxysteroid dehydrogenase type 2 (3*β*HSD II) in granulosa cells [35]. We also demonstrated that PPARs and their steroidogenic targets are influenced by the presence and source of LH in the ovarian stimulation protocol. These data revealed a key role of PPARs in the ovarian follicle growth and competence and widen the knowledge on the effects of ovarian stimulation protocol on ovarian physiology. In addition, it is well known that the action of cyclooxygenase 2- (COX2-) derived prostacyclins which are factors essential for embryo development, hatching, and implantation [30, 31] is mediated by PPAR*β*/*δ*. Indeed, blastocyst hatching is reduced in PPARb null mice and a beneficial effect of prostacyclin is observed in WT embryos and embryos exposed to a synthetic PPAR*β*/*δ* ligand [78]. Therefore, PPAR*β*/*δ* may be considered as novel therapeutic target to improve IVF outcome.

An important effect of PPARs on steroidogenesis is related to the ability of PPAR*γ* to counteract the interaction of NF-*κ*B with the aromatase promoter II [20] by inhibiting the expression of P-450 (CYP19A1), also known as aromatase or estrogen synthase, the rate limiting enzyme for the conversion of androgens to estradiol. In a recent study by Rak-Mardyła and Karpeta [21], it was observed in porcine ovarian follicles that rosiglitazone significantly increases PPAR*γ* expression, progesterone secretion, 3*β*HSDII activity, and protein expression but decreases androstenedione and testosterone secretion by reducing the expression and activity of 17-hydroxylase, 17,20-lyase (CYP17), and 17*β*-hydroxysteroid dehydrogenase (17*β*HSD) and had no effects on estradiol secretion and CYP19A1 activity. PPAR*γ* can also affect steroidogenesis by decreasing the production of androgenic precursors in the theca cells, antagonizing the stimulation of androstenedione, typically induced by LH/insulin combination in disorders such as PCOS, hyperinsulinism, oligoovulation, and hirsutism [79]. Veldhuis and colleagues demonstrated that synthetic thiazolidinedione (troglitazone) and a natural ligand of PPAR*γ* impede the concerted stimulation by LH and insulin of* in vitro* thecal cell androgen production, CYP17 gene expression, and CYP17 protein phosphorylation. In porcine ovaries resistin, the adipose tissue-specific secretory factor (ADSF) associated with ovarian dysfunction in obese women is known to upregulate PPAR*γ*, which, in turn, reduces resistin expression. GW9662, a synthetic PPAR*γ* antagonist, was capable of reversing the effect of resistin on steroid hormone secretion [80]. In mice, the ovarian tissue-specific deletion of PPAR*γ* led to subfertility, principally due to a decreased number of implanted embryos and a decreased progesterone secretion by the corpus luteum [81].

### 3.3. Uterus and Placenta

The expression of all PPAR isotypes has been reported in uterus and placenta and a correlation between PPARs expression profiles in uterus and the development of placenta has been demonstrated [82]. A species-specific expression profile of PPAR isotypes has been observed. In sheep endometrium, PPAR*α* is mainly expressed during early stages of pregnancy with a decrease between day 7 and day 17. PPAR*β*/*δ* is constantly expressed during pregnancy, whereas PPAR*γ* expression is irregular [13]. In addition in this animal model, the expression of PPARs seems to be unrelated to the expression of RXRs, probably due to different heterodimer composition [83]. In rodents, PPAR*α* and PPAR*β*/*δ* are expressed in the placenta labyrinth and junctional zone, whereas in humans, they are observed in villous trophoblasts and in particular in syncytiotrophoblasts [84]. In the rat, the expression of PPAR*β*/*δ* is decreased in endometrial stromal cells during implantation [85]. PPAR*γ* was also detected in mouse [86] and rat placenta [87] by days 8 and 11, respectively. In human placenta, PPAR*γ* is expressed in early and full-term villous trophoblasts and in extravillous trophoblasts in first-trimester placentas [88]. In mice PPAR*γ* is expressed in spongiotrophoblasts, in the vascular labyrinth necessary for maternal and fetal nutrient exchange. In cultured villous trophoblasts of human term placenta, PPAR*α* and PPAR*β*/*δ* transcript levels were higher in cytotrophoblasts than in syncytiotrophoblasts [89]. PPAR*γ*1 is the only PPAR*γ* isoform clearly detectable in dog uterine/placental tissues. Placental expression of PPAR*γ* is elevated after implantation and at midgestation, followed by a prepartal downregulation. All changes are more pronounced at the protein level suggesting that the PPAR*γ* expression may be regulated at posttranscriptional level [90].

*PPARa*-knockout mice are associated with increased rates of maternal abortion, although surviving pups develop normally, while* PPARg*- and* PPARb*-knockout mice are embryonic-lethal due to gross placental abnormalities and defects in placental morphogenesis, respectively [91].

PPAR*β*/*δ* null mutant mice showed defects in placenta development, probably due to the involvement of PPAR*β*/*δ* in differentiation of trophoblast giant cells [92]; in addition PPAR*β*/*δ* is also known to regulate the production of COX2-derived prostacyclin I2, which enhances mouse blastocyst invasion [78]. Moreover PGI2-induced PPAR*β*/*δ* activation accelerates blastocyst hatching in mice [31].

PPAR*γ in vivo* and* in vitro* studies indicate that inactivation of PPAR*γ* leads to defective trophoblast differentiation, early embryonic lethality, and severe developmental placental damage, which together can affect the placental labyrinth zone development [93–95]. For instance, PPAR*γ* activity affects trophoblast differentiation by regulating the functions of both villocytotrophoblast (VCT) and extravillocytotrophoblast (EVCT) [91].

In conclusion we can assert that PPARs play important roles as key-factors responsible for controlling the process of placental development (for more details see review by Meher et al., 2015 [96]).

### 3.4. Mammary Glands

All three isotypes of PPAR are detected in rodent mammary gland and human breast cell lines [97, 98]. During pregnancy and lactation, PPAR*α* and PPAR*γ* mRNAs decreased while PPAR*β*/*δ* mRNA remained relatively unchanged in mouse mammary gland [97]. Changes in PPAR*γ* and endocrine-related genes in perfluorooctanoic acid (PFOA) treated mice delay mammary gland development at weaning [99]. PPAR*γ* adipose tissue-specific knockout mice exhibit absence of dermal fat and absence of mammary gland development with loss of mammary fat pads, suggesting a critical role for PPAR*γ* mammary gland development [100]. Moreover, its role in regulating the triacylglycerol synthesis and secretion in goat mammary cells outlines the functional importance of PPAR*γ* in mammary gland tissue during lactation [101]. PPAR*γ* loss in mammary secretory epithelial (MSE) cells results in decreased expression of PTEN and other factors contributing to a protumorigenic environment [102].

## 4. PPAR Implications in Subfertility

Female fertility and energy metabolism are tightly interconnected. During the reproductive life, the physiological activity of the gonads, with their cyclic production of sex hormones, ensures a continuous regulation of food intake and energy expenditure. In female mammals, alterations of gonadal activity, including age-dependent decrease of ovarian functions, are associated with disruption of metabolic homeostasis and consequent inflammatory reactions that trigger the onset of metabolic, cardiovascular, skeletal, and neural pathologies [103]. PCOS is a major metabolic and reproductive disorder characterized by hyperandrogenism, chronic anovulation, and polycystic ovaries [104], as well as metabolic disturbances. Half of PCOS patients have overweight or obesity [105] and dyslipidemia is also commonly observed [106]. Given the role of PPARs in regulating energy homeostasis and carbohydrate metabolism, there is an increasing interest in the use of its agonists for the restoration of metabolic homeostasis in women with ovarian dysfunction [107, 108]. Synthetic PPAR agonists are used for therapeutic treatment of metabolic diseases, including dyslipidemia [109], insulin resistance [110], and type 2 diabetes [111]. Particularly, fibrates, well-known PPAR*α* agonists, are commonly prescribed for the treatment of dyslipidemia [112]. Several TZDs, structural analogues of fibrates including pioglitazone and rosiglitazone, improve glycemic control in patients with type 2 diabetes or glucose intolerance via their insulin-sensitizing activity, mainly achieved by decreasing circulating fatty acids levels and increasing glucose uptake in insulin-sensitive tissues, like skeletal muscle. Current data show that TZDs can effectively reduce insulin and fasting blood glucose levels in patients with PCOS, but they do not effectively reduce androgen levels and seem to increase body weight [113]. A further study has reported that low-dose therapeutic regimen with rosiglitazone and metformin, a hypoglycemic drug used in the treatment of diabetes, has comparable beneficial impacts on metabolic, hormonal, and morphological features of PCOS [114]. Positive effects of pioglitazone, a PPAR*γ* activator, on carnitine metabolism and insulin sensitivity in PCOS patients have been described [115]. Theca cells are the sole ovarian site of synthesis of DHEA, which is a precursor of androstenedione, an essential ligand for PPAR*α*, and DHEA supplementation improves follicular microenvironment in poor responder patients [76]. Recent data have reported that administration of Irbesartan (IRB), a PPAR*γ* agonist, during chemotherapy alleviates the ovarian toxicity by cyclophosphamide (CPM), a chemotherapeutic agent. IRB coadministration restored ovarian antioxidant defences, such as increased glutathione (GSH) levels and superoxide dismutase (SOD) activity and decreased levels of malondialdehyde (MDA), an indicator of lipid peroxidation. In addition, the increase in E2 and reduction in FSH levels observed in IRB rats support the hypothesis that ovarian function is protected by activation of PPAR*γ* signalling contributing to CPM detoxification and antioxidant response [116] that can be explored for future implications in oncofertility. In spite of the role of PPARs in ovarian homeostasis, it is important to highlight that toxic compounds such as industrial plasticizers, herbicides, and organic solvents can act as powerful PPAR agonists [8]. Unintended exposure to chemicals having PPAR*α* and PPAR*γ* agonistic activities induces notable toxicological results, such as endocrine disruption [117]; particularly mono(2-ethylhexyl) phthalate MEHP is distinct from several structurally related phthalates but similar to the peroxisome proliferator Wy-14,643 in its action on granulosa cell estradiol production.

Among various environmental chemicals, MEHP, a metabolite of dioctyl terephthalate bis(2-ethylhexyl) benzene-1,4-dicarboxylate (DEHP), transactivates PPAR*α* and PPAR*γ* in both human and mouse [118]; the triphenyltin, used as fungicide, acts as a ligand of PPAR*γ*  [119]; diclofop-methyl and pyrethrins, two common pesticides, induce PPAR*α* agonistic activity* in vivo* as well as* in vitro* [120].

In a recent study the effect of the exposure to DEHP on ovarian expression of estrogen receptor *α* (Esr1) and aromatase (Cyp19a1) in three generations of Sv/129 wild-type (WT, +/+) and PPAR*α* (−/−) knockout mice is investigated. Ovarian expression of Esr1 decreases in response to DEHP treatment in all WT generations, but in PPAR*α* null mice this treatment was ineffective, suggesting that transgenerational repression of ovarian Esr1 gene expression by DEHP is mediated by PPAR*α*-dependent pathways [121].

## 5. Future Perspective

In conclusion, a large number of studies have demonstrated that PPARs might play an important role in female reproduction, including ovarian function, gestation, and communication between mother and fetus. The use of synthetic PPAR ligands employed to ameliorate complications related to metabolic disorders has recently been expanded to disorders in the reproductive area. Moreover, the correlation between PPARs and steroidogenesis during follicular differentiation supports further research aimed at understanding if controlled ovarian hyperstimulation in IVF cycles may benefit from their pharmacological manipulation. Nevertheless, research in this field needs to be associated with the knowledge of mechanisms underlying deleterious effects on fertility of uncontrolled exposure to PPAR synthetic ligands.

In conclusion, PPARs are new players in the field of reproduction being expressed and functional in a variety of animal reproductive tissues. In addition, from studies with PCOS women [115, 116], it is now clarified that ligands of PPARs ameliorate fertility. However, the knowledge on expression and function of PPARs and its ligands in other diseases linked to infertility and disturbed pregnancy is limited and deserves further investigations, especially in humans.

## Abbreviations

ADSF: Adipose tissue-specific secretory factor
COS: Controlled ovarian stimulation
COX2: Cyclooxygenase 2
CPM: Cyclophosphamide
CYP17: Cytochrome P450 17-alpha-hydroxylase/17,20-lyase
CYP19A1: Cytochrome P-450
DEHP: Di-2-ethylhexylphthalate
E2: 17b-estradiol
ERE: Estrogen response element
Esr1: Estrogen receptor *α*
EVCTS: Extravillous cytotrophoblast
Fshb: Follicle stimulating hormone, beta polypeptide
FSH: Follicle stimulating hormone
GnRH: Gonadotropin realising hormone
hCG: Human CG
HMG-CoA red: 3-Hydroxy-3-methylglutaryl coenzyme A reductase
hMG-HP: Highly purified human menopausal gonadotropin
3*β*-HSD: 3*β*-Hydroxysteroid dehydrogenase
3*β*HSD II: 3*β*-hydroxysteroid dehydrogenase
17*β*HSD IV: 17*β*-Hydroxysteroid dehydrogenase type IV
IRB: Ibersartan
IVF: * In vitro* fertilization
LH: Luteinizing hormone
Lhb: Luteinizing hormone beta polypeptide
LH/CG: Luteinizing hormone/choriogonadotropin
MDA: Malondialdehyde
MEHP: Mono(2-ethylhexyl) phthalate
MSE: Mammary secretory epithelial
NF-*κ*B: Nuclear Factor Kappa B
NR1H3: Nuclear receptor subfamily 1, group H, member 3
PCOS: Polycystic ovary syndrome
PFOA: Perfluorooctanoic acid
PG: Prostaglandin
PGI2: Prostacyclin
PKO: Perforin knockout
PMSG: Pregnant mare's serum gonadotropin
PPRE: PPAR-responsive element
PTGS2: Prostaglandin-endoperoxide synthase 2
r-hFSH: Recombinant human follicle stimulating hormone
r-hLH: Recombinant human luteinizing hormone
SCARB1: Scavenger receptor class B member 1
SOD: Superoxide dismutase
STAR: Steroidogenic acute regulatory protein
TZDs: Thiazolidinediones
VCTs: Villous cytotrophoblasts.
